# Molecular Biology: Estrogens Shield Breast Cancer Cells

**DOI:** 10.1289/ehp.115-a297a

**Published:** 2007-06

**Authors:** M. Nathaniel Mead

Among the more insidious aspects of cancer is its capacity for escaping the anti-cancer defenses of the host. New research suggests that some estrogens may further reinforce this evasion of host immunity, even as those same hormones stimulate the growth and spread of hormone-responsive cancers. According to David Shapiro, a medical and biochemistry professor at the University of Illinois at Urbana–Champaign, the new findings highlight the role that estrogen-related interference with immune cell function may play in the development and progression of breast cancer.

Shapiro and his colleagues, reporting in a paper posted 22 January 2007 ahead of publication in *Oncogene*, observed that estrogen induces the expression of a newly discovered gene in humans for proteinase inhibitor 9 (PI-9). When estrogen binds to receptors in the cancer cell, the resulting complex promotes production of the PI-9 protein, which in turn binds granzyme B, the primary protease used by natural killer (NK) cells to kill off transformed or infected cells.

NK cells normally play a central role in immune surveillance against the metastatic spread of cancer. Previous research out of Leiden University Medical Center in the Netherlands, published in the 25 September 2001 issue of *Proceedings of the National Academy of Sciences*, had shown that increased levels of PI-9 in some tumors and tumor-derived cell lines may enhance these tumors’ ability to evade apoptosis mediated by NK cells and cytotoxic T cells.

In the current paper, the researchers linked increasing concentrations of estrogen with increasing levels of PI-9 and progressively blocked cell death by NK cells. “The levels of estrogen required to induce [PI-9] in breast cancer cells are extremely low,” says Shapiro. Moreover, at elevated levels of epidermal growth factor and estrogen receptor–α, the induction of PI-9 by either estradiol or the breast cancer drug tamoxifen effectively blocked the killing of cancer cells by NK cells. Levels of epidermal growth factor are elevated in many women with breast cancer, and this adversely affects the prognosis. The presence of estrogen receptor–α at the time of diagnosis is an indication for using tamoxifen or other forms of endocrine therapy.

Tamoxifen is known to have mixed agonist/antagonist effects on estrogen, stimulating uterine cancer at low doses while inhibiting this and other estrogen-responsive cancers at higher doses. In contrast, the drug raloxifene, which is commonly used to prevent osteoporosis and has also been shown to reduce the risk of some breast cancers, had no immune-disrupting effects.

Shapiro’s findings come with the following caveat: most estrogen-responsive breast tumors contain low to moderate levels of estrogen receptors, and in these tumors tamoxifen will not induce a level of PI-9 that enables the breast cancer cells to evade killing by immune cells. Only in the relatively small subset of breast tumors that contain very high levels of estrogen receptors will tamoxifen have an effect that could effectively shield the cancer from attacking immune cells.

Indeed, a 21 June 2006 *JAMA* study—a prospective, double-blind, randomized clinical trial conducted in nearly 200 cancer treatment centers throughout North America—directly compared tamoxifen and raloxifene, and found both drugs to be chemopreventive. “In that large trial, tamoxifen even [seemed to perform] better in the control of noninvasive breast cancers,” says V. Craig Jordan, scientific director for the Fox Chase Cancer Center Medical Science Division. “So while these [University of Illinois] data are important and insightful, we should not lose sight of what the clinical data are telling us.”

Estrogenic compounds such as DDT and dioxins may also impede immune protection against breast cancer cells. “The mechanism that this study provides for estrogen-mediated decreases in NK cell function may also be operative in the decreases in NK function that have been observed with certain environmental estrogens, such as DDT,” says Margaret Whalen, an associate professor of chemistry at Tennessee State University who has studied the immune-suppressive effects of various organochlorine pesticides. Most human exposures to xenoestrogens involve mixtures at relatively low levels, Whalen adds, and concurrent exposure to multiple chemicals may alter the immunotoxicity of a particular chemical.

Shapiro and his colleagues are now looking into the factors that contribute to regulation of PI-9 in postmenopausal women. “Blocking the production of this protein represents a potential new target for breast cancer therapies,” he says.

